# Interrelations of disease activity, health-related quality of life, and mental health in axial spondyloarthritis: the Rheuma-VOR cohort

**DOI:** 10.3389/fmed.2026.1823684

**Published:** 2026-05-13

**Authors:** Lina Judit Schiestl, Eva Wendt, Fabian Proft, Kirsten Hoeper, Torsten Witte, Gunter Assmann, Andreas Schwarting, Matthias Dreher

**Affiliations:** 1 Division of Rheumatology and Clinical Immunology, Department of Internal Medicine I, University Medical Center of the Johannes Gutenberg University, Mainz, Germany; 2 Department of Gynecology and Obstetrics, University Medical Center of the Johannes Gutenberg University, Mainz, Germany; 3 Department of Internal Medicine I, University Medical Center Kiel, Kiel, Germany; 4 Department of Gastroenterology, Infectiology and Rheumatology (including Nutrition Medicine), Charité – Universitätsmedizin Berlin, corporate member of Freie Universität Berlin and Humboldt-Universität zu Berlin, Berlin, Germany; 5 Center for Rheumatology Lower Saxony, Hannover e.V, Hannover, Germany; 6 Department of Rheumatology and Immunology, Hannover Medical School, Hanover, Germany; 7 Center of Rheumatology and Clinical Immunology, Ruhr-University Bochum, Bochum, Germany; 8 Center for Rheumatology Rhineland-Palatinate, Bad Kreuznach, Germany; 9 University Center for Autoimmunity, University Medical Center of the Johannes Gutenberg University, Mainz, Germany

**Keywords:** axial spondyloarthritis, disease activity, early diagnosis, health-related quality of life, mental health

## Abstract

**Background:**

Axial spondyloarthritis (axSpA) is a chronic inflammatory rheumatic disease characterized by pain and stiffness of the axial skeleton, peripheral manifestations like arthritis, dactylitis and enthesitis, extra-musculoskeletal manifestations, and reduced health-related quality of life (HRQoL). Depressive symptoms and fatigue are common, yet few studies have assessed these outcomes at diagnosis and during early treatment.

**Objective:**

To evaluate mental health, fatigue, HRQoL, and their association with disease activity and functional status in patients with axSpA at diagnosis and after 1 year of rheumatologic care.

**Methods:**

Rheuma-VOR is a multicenter, proof- of concept study in Germany implementing structured preselection and early referral for suspected axSpA. We included 238 patients with confirmed axSpA, of whom 76 completed a 12-month follow-up. Disease activity (BASDAI, ASDAS), functional status (BASFI, BASMI, FFbH), mental health (PHQ-9, WHO-5), fatigue (FACIT-F), and HRQoL (EQ-5D) were assessed at baseline and follow-up. Associations between disease activity, function, and patient-reported outcomes (PROs) were analyzed using correlation and multivariable regression.

**Results:**

At diagnosis, patients exhibited high disease activity (BASDAI 4.6 ± 2.0, ASDAS 2.6 ± 0.9) and substantial prevalence of depressive symptoms (PHQ-9 ≥ 10 in 36.5%) and fatigue (FACIT-*F* < 39 in 69.5%). One-year follow-up showed significant improvements in disease activity, functional impairment, HRQoL, mental well-being, and fatigue (all *p <* 0.05). Higher patient-reported disease activity (BASDAI) consistently predicted depressive symptoms and fatigue, whereas functional capacity (FFbH) was the strongest predictor of HRQoL. Physician-assessed disease activity (ASDAS) and functional impairment (BASFI) had smaller or time-limited effects.

**Conclusion:**

In axSpA, patient-reported disease activity and functional capacity are key determinants of mental health, HRQoL, and fatigue. Early diagnosis and initiation of guideline-concordant therapy are associated with improvements across physical and psychological domains, supporting systematic screening and interdisciplinary management strategies.

## Introduction

Axial spondyloarthritis (axSpA) is a chronic, immune-mediated inflammatory rheumatic disease characterized by a progressive disease course and the absence of curative treatment options (1, 2). Disease onset typically occurs at a young age, most frequently before the age of 45 years (3). AxSpA predominantly affects the axial skeleton, with inflammatory involvement of the sacroiliac joints (SIJs) representing the hallmark of early disease; however, it may also present with peripheral manifestations, including arthritis, enthesitis, and dactylitis. From a pathophysiological perspective, persistent inflammation at the entheses and within the synovium contributes to structural damage, including the development of new bone formation and ankylosis of the spine (4).

The clinical phenotype of axSpA is heterogeneous and extends beyond axial musculoskeletal manifestations. In accordance Assessment of SpondyloArthritis International Society (ASAS) definitions, extra-musculoskeletal manifestations are uveitis, psoriasis and inflammatory bowel disease. Common comorbidities are cardiovascular involvement, depression and fatigue (5, 6).

Given the high prevalence of musculoskeletal symptoms in the general population-often attributable to degenerative changes, mechanical stress, physical inactivity, or psychosocial factors—early recognition of axSpA in primary care remains challenging (7). Nevertheless, timely diagnosis is of major clinical relevance, as early initiation of effective therapy is associated with improved disease outcomes and may limit structural progression (8). Despite this, the mean diagnostic delay from symptom onset to established diagnosis remains approximately 6–8 years (9, 10). On the other hand, prolonged exposure to inflammatory back pain, diagnostic uncertainty, and functional limitations are frequently associated with impaired mental health (11–13).

Depressive symptoms represent a common comorbidity in axSpA and are often accompanied by additional disease-related or treatment-related comorbidities. Epidemiological studies indicate that approximately 25% of patients with axSpA report clinically relevant depressive symptoms (13–15).

Health-related quality of life (HRQoL) is a multidimensional outcome measure influenced by multiple interacting determinants. The literature consistently identifies three major domains affecting HRQoL in axSpA: physical functioning and disease activity, mental health, and socioeconomic factors. Consequently, patients with axSpA report substantially reduced HRQoL compared with the general population (16–18). Patients with high disease activity are particularly affected and report an increased prevalence of depressive symptoms (19, 20). In addition, fatigue represents a highly prevalent and clinically relevant symptom in axSpA that substantially contributes to impaired HRQoL. The main target of the Rheuma-VOR study was an early diagnosis of inflammatory joint diseases and therefore axSpA through standardized screening procedures (9). Rapid referral for appropriate diagnostic work-up and timely diagnostic confirmation enabled initiation of guideline-concordant therapy at a median of 3.92 years in comparison to 8.41 years analyzed in a matched standard care cohort of the German Rheumatism Research Centre after symptom onset (9, 21–23).

To our knowledge, this study is among the few to comprehensively assess both mental health and HRQoL at the time of initial diagnosis and following the initiation of therapy in patients with axSpA.

## Materials and methods

### Study design and setting

Rheuma-VOR is a multicenter, proof-of-concept study conducted in Germany. The central aim of the program was to reduce diagnostic delay in patients with inflammatory joint diseases by shortening the interval between symptom onset, rheumatologic assessment, and confirmed diagnosis. To achieve this, a structured preselection strategy using standardized screening instruments, coordinated patient pathways, and optimized referral procedures were implemented across the 72 participating rheumatology centers and practices in four federal states in Germany (Berlin, Lower Saxony, Rhineland-Palatinate, and Saarland). Importantly, the study was designed to facilitate earlier diagnosis, while maintaining guideline-compliant therapy. Accordingly, after confirmation of axSpA, all patients received routine guideline-conform standard-of-care management according to contemporary national and ASAS-EULAR recommendations, at the discretion of the treating rheumatologist (23).

Inclusion took place between August 2017 and December 2020, and data registration and collection were performed within the same period. As part of this prospective observational study, questionnaire-based patient-reported outcomes were collected longitudinally. Owing to the predefined study end date (December 31, 2020), patients enrolled after January 1, 2020, could not complete the one-year follow-up period and were therefore not eligible for the longitudinal follow-up analyses (9, 22).

### Study population

A total of 238 patients with a confirmed diagnosis of axSpA were included. Patients were identified in primary care using a standardized screening questionnaire. Screening forms were reviewed by a central coordination center. Patients with a high likelihood of axSpA were referred to a board-certified rheumatologist for further diagnostic evaluation. Diagnostic confirmation was performed by the treating rheumatologist in accordance with established clinical standards, guided by the ASAS classification criteria for axSpA. Patients received treatment according to current clinical guidelines and at the discretion of the treating physician (23). Approximately one third of the cohort participated in a follow-up assessment after 12 months.

### Study procedures and data collection

Baseline assessment was conducted at the rheumatology visit following diagnostic confirmation and initiation of therapy. Follow-up assessment was performed after 12 months. At both time points, data were collected on sociodemographic characteristics, medical history, disease activity, functional status, mental health, and HRQoL using physician-assessed measures and patient-reported outcome instruments. Completion of patient-reported questionnaires was supported by trained healthcare personnel, predominantly nurses, to ensure consistency and to account for potential differences in educational and socioeconomic backgrounds.

### Disease activity and functional status

Disease activity was assessed by the treating rheumatologist and quantified using validated patient-reported outcome measures, including the Bath Axial Spondylitis Disease Activity Index (BASDAI) (24) and the Axial Spondylitis Disease Activity Score (ASDAS) (25). Functional status and spinal mobility were evaluated using the Bath Axial Spondylitis Metrology Index (BASMI) (26), the Functional Questionnaire Hannover (FFbH) (27), and the Bath Axial Spondylitis Functional Index (BASFI) (28).

### Mental health and health-related quality of life

Mental health outcomes were assessed using validated patient-reported outcome measures. Depressive symptoms were measured using the Patient Health Questionnaire-9 (PHQ-9) (29) and the World Health Organization-5 Wellbeing Index (WHO-5) (30). HRQoL life was assessed using the EuroQol 5-Dimension questionnaire (EQ-5D) (31). Fatigue was assessed using the Functional Assessment of Chronic Illness Therapy–Fatigue scale (FACIT-F) (32).

### Statistical analysis

Statistical analyses were conducted in accordance with STROBE recommendations for observational studies (33). Continuous variables are presented as means with standard deviations or medians with interquartile ranges, as appropriate. Categorical variables are presented as frequencies and percentages.

For the present analyses, two predefined analytical samples were considered. The full baseline cohort (*n =* 238) was used for exploratory cross-sectional analyses. In addition, the subgroup of patients with complete one-year follow-up data (*n =* 76) was used for longitudinal analyses. Group comparisons were performed using independent *t*-tests or Mann–Whitney U tests, depending on data distribution. For dependent groups (Baseline and Follow-up) *t*-tests or Kruskall-Wallis test were used. Associations between variables were examined using Sperman’s correlation analyses. To further investigate independent associations, multivariable linear regression analyses were performed. The dependent variables were mental health (PHQ-9 and WHO-5), HRQoL (EuroQoL-5D) and Fatigue (FACIT-F) with disease activity scores included as the independent variables. Potential covariates, including body mass index (BMI) and diagnostic delay, were explored in preliminary analyses but were not included in the final model due to lack of significant association with the outcome variable.

All statistical tests were two-sided, and a *p* < 0.05 was considered statistically significant. Statistical significance levels in the tables are indicated as follows: *p <* 0.05 (*), *p <* 0.01 (**).

All analyses were performed using SPSS software (IBM Corp.), version 29. In addition, R software was used to generate graphical visualizations for the heat map for correlation analysis.

## Results

During the study period 238 patients with confirmed axSpA were enrolled. A total of 76 patients (31.8%) completed both baseline and 12-month follow-up and were included in longitudinal analyses.

The total cohort at baseline refers to all patients initially included in the study, whereas V1 follow-up represents the longitudinal subset of patients who completed the one-year follow-up assessment, and V2 the corresponding follow-up visit. At baseline (total cohort), the mean age was 39.3 (± 13.4) years; 46% were male. The mean BMI was 27.2 (± 5.6) kg/m^2^; 36.8% were current smokers, and 44.9% reported ≥1 h/week of physical activity.

With respect to disease history, the mean age at symptom onset was 35.0 years (SD ± 13.5). The median duration of symptoms prior to diagnosis was 19.0 months (mean duration: 52.5 months SD ± 72.5 months). HLA-B27 positivity was observed in 142 patients (59.4%). The mean number of reported comorbidities was 3.5 (SD ± 3.0).

Analyses of sociodemographic variables, including gender, marital status, and employment status, revealed no statistically significant associations with the outcomes of interest; therefore, these variables were not included in the main results.

Baseline sociodemographic and clinical characteristics are summarized in Table 1.

**Table 1:** Baseline characteristics at study inclusion.

Baseline characteristics at study inclusion	Values total cohort visit 1 (*n =* 238)	Values visit 1 follow-up cohort (*n =* 76)
Sociodemographic characteristics		
Age, years	Mean: 39.3 (± 13.4)	Mean: 38,64 (±12.7)
Sex		
Male	110	37
Female	128	39
Living situation		
Living alone	40 (16.8%)	11 (14.5%)
Employment status		
Unemployed	29 (12.2%)	9 (11.8%)
Body mass index (BMI)	Mean: 27.2 kg/m^2^ (± 5.6)	Mean: 28.13 kg/m^2^ (±5.9)
Current smoking	88 (37.0%)	32 (42.1%)
Physical activity (>1 h/week)	106 (44.6%)	37 (48.7%)
Disease history		
Age at symptom onset, years	Mean: 35.0 (± 13.5)	Mean: 35.0 (± 12.3)
Symptom duration until diagnosis, years	Mean: 3.92 (± 6.0)	Mean 3.2 (± 4.9)
HLA-B27 positive	142 (59.7%)	43 (56.6%)
Number of comorbidities	3.5 (± 3.0)	4.0 (± 3.2)

n = number of included patients; h = hour; HLA-B27 = human Leukocyte Antigen-B.

### Disease activity and functional outcomes at baseline and 1 year follow-up

At baseline, patients exhibited high disease activity (BASDAI 4.6 ± 2.0; ASDAS 2.6 ± 0.9). 61.4% (BASDAI) and 67.0% (ASDAS) of included patients met criteria for high disease activity. Functional capacity (FFbH 80% ± 16.7) was preserved in most patients, with only 12.8% showing clinically relevant impairment. BASFI (3.1 ± 2.4) and BASMI (1.2 ± 1.7) indicated moderate functional limitation and spinal mobility impairment.

After 1 year of rheumatologic treatment, disease activity showed a statistically significant improvement across all disease activity measures. Both ASDAS and BASDAI decreased significantly between baseline (V1) and follow-up (V2).

Mean ASDAS decreased from 2.6 (SD ± 0.9) at baseline to 2.3 (SD ± 0.9) at follow-up, corresponding to a mean change (ΔV1–V2) of 0.35 (SD ± 1.1; *p =* 0.009). Similarly, BASDAI improved from a mean of 4.9 (SD ± 2.0) at baseline to 3.8 (SD ± 2.1) at follow-up, with a mean reduction of 1.05 points (SD ± 2.0; *p <* 0.001).

Functional impairment (BASFI), also improved significantly over time. Mean BASFI decreased from 3.3 (SD ± 2.4) at baseline to 2.6 (SD ± 2.3) at follow-up, resulting in a mean change of 0.71 (SD ± 2.3; *p =* 0.010). Spinal mobility showed a statistically significant improvement over the observation period. BASMI decreased from 1.3 (SD ± 1.8) at baseline to 0.9 (SD ± 1.9) at follow-up, corresponding to a mean change of 0.48 (SD ± 2.2; *p =* 0.007).

In contrast, functional capacity in daily life, as measured by the FFbH, improved numerically but did not reach statistical significance.

Detailed results for disease activity, functional status, and mobility at baseline and follow-up are presented in Table 2.

**Table 2:** Disease activity, functional status, and mobility at baseline and follow-up.

Measurement Instrument	Total cohort* n = 238 MW (± SD)	V1* n = 76 MW (± SD)	V2* n = 76 MW (± SD)	V1 – V2 ΔV1-V2
ASDAS	2.6 (± 0.9)	2.6 (± 0.9) M: 2.52 (±0.9) F: 2.68 (±0.9) *p =* 0.22	2.3 (±0.9) M: 2.24 (±0.9) F: 2.4 (± 0.9) *p =* 0.5	*t*-Test ***p =* 0.009** **Δ 0.35 (±1.1)**
BASFI	3.1 (± 2.4)	3.3 (± 2.4) M: 3.02 (±2.4) F. 3.17 (±2.4) *p =* 0.66	2.6 (±2.3) M: 2.45 (±2.3) F: 2.64 (±2.4) *p =* 0.72	WCX ***p =* 0.010** **Δ 0.71 (±2.3)**
BASDAI	4.6 (± 2.0)	4.9 (± 2.0) M: 4.4 (±2.0) F: 4.7 (±2.0) *p =* 0.19	3.8 (±2.1) M: 3.56 (±2.2) F: 4.04 (±2.0) *p =* 0.33	WCX ***p <* 0.001** **Δ 1.05 (±2.0)**
BASMI	1.2 (± 1.7)	1.3 (± 1.8) M: 1.37 (±2.0) F: 1.00 (±1.5) *p =* 0.12	0.9 (±1.9) M: 1.2 (±2.3) F: 0.6 (±1.2) *p =* 0.22	WCX ***p =* 0.007** **Δ 0.48 (±2.2)**
FFbH	80 (±16.7)	80.2 (±16.7) M: 80.58 (±18.2) F: 80.02 (±15.5) *p =* 0.8	83.7 (±16.9) M: 84.24 (±17.7) F: 83. 23 (±16.4) *p =* 0.8	WCX *p =* 0.091 Δ − 3.6 (±15.3)

^*^Total cohort baseline includes all enrolled patients; V1 comprises only patients with available 1-year follow-up data (V2). M: male; F: female.

### Mental well-being and health-related quality of life

At baseline, mental well-being assessed by WHO-5 showed a mean score of 43.5 (SD ± 30.0). According to established WHO-5 cut-offs, depression was classified as unlikely in 51.3% of patients, mild depressive symptoms were observed in 15.8%, and values indicative of major depression were observed in 32.9% of the cohort. Consistently, the mean PHQ-9 score was 8.4 (SD ± 5.2), corresponding to mild or subthreshold depressive symptomatology. Based on PHQ-9 categories, 30.5% of patients showed no depressive symptoms, while 36.5% met criteria for major depressive symptoms. The remaining patients were classified as having mild to moderate depressive symptoms.

The mean FACIT-F score at baseline was 33.3 (SD ± 11.1). According to FACIT-F thresholds, 30.5% of patients showed no indication of fatigue, while 69.5% were classified as having mild to severe fatigue.

HRQoL assessed by the EuroQol-5-Dimension questionnaire (EQ-5D) yielded a mean index value of 0.7 (SD ± 0.3) at baseline.

At follow-up, statistically significant differences between baseline (V1) and follow-up (V2) were observed for all four scores. EQ-5D index values differed between V1 and V2 (mean difference −0.085, SD ± 0.25; Wilcoxon signed-rank test, *p =* 0.006). WHO-5 scores differed between V1 and V2 (mean difference −8.5, SD ± 23.1; *p =* 0.015). PHQ-9 scores differed between V1 and V2 (mean difference 2.0, SD ± 4.5; *p <* 0.001). FACIT-F scores differed between V1 and V2 (mean difference −4.0, SD ± 9.4; *p =* 0.001).

Baseline and follow-up values are presented in Table 3.

**Table 3:** Baseline and follow-up health-related quality of life and mental health.

Measurement Instrument	Total cohort *n =* 238 Mean (± SD)	V1 *n =* 76 Mean (± SD);	V2 *n =* 76 Mean (± SD);	V1 – V2 ΔV1-V2
EuroQoL-5D	0.7 (± 0.3)	0.7 (± 0.3)	0.8 (±0.3)	WCX *P =* 0.006 Δ − 0.085 (±0.25)
WHO-5	43.5 (± 30)	41.7 (± 25)	50.9 (±26.6)	WCX *P =* 0.015 Δ − 8.5 (±23.1)
PHQ-9	8.4 (± 5.2)	9.1 (±5.4)	7.0 (±4.9)	WCX *P <* 0.001 Δ2.0 (±4.5)
FACIT-F	33.3 (± 11.1)	31.9 (±11)	35.4 (±10.1)	WCX *P =* 0.001 Δ-4.0 (±9.4)

### Associations between disease activity, function, and patient-reported outcomes

**Figure 1:**
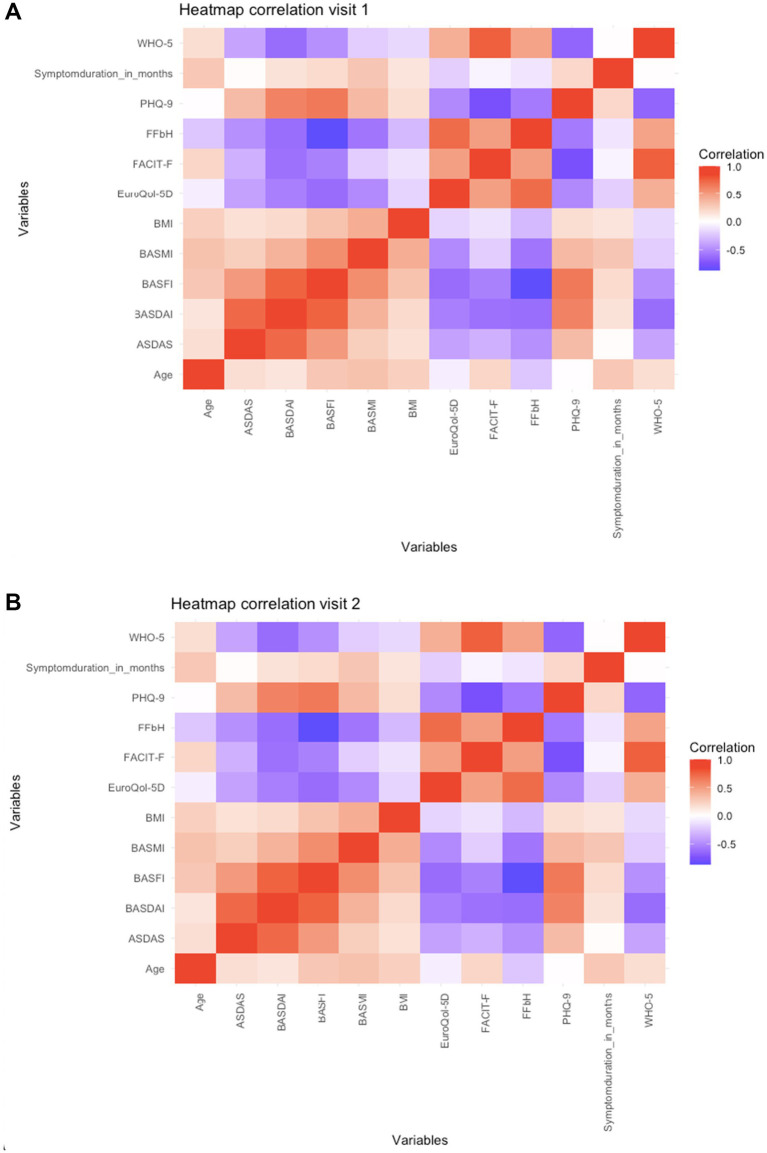
**(A)** Heatmap correlation visit 1. **(B)** Heatmap correlation visit 2.

At both time points, higher disease activity (ASDAS, BASDAI) and greater functional impairment (BASFI) were consistently associated with worse mental well-being (WHO-5), lower HRQoL (EQ-5D), higher depressive symptoms (PHQ-9), and greater fatigue (FACIT-F). Given the opposite scoring directions of the instruments, with higher BASDAI values indicating greater disease activity and lower WHO-5 and FACIT-*F* values indicating poorer mental health and more severe fatigue, respectively, the correlations with BASDAI were inverse in direction. In contrast, higher functional capacity (FFbH) correlated with better mental health and HRQoL, and lower depressive symptoms and fatigue. In an additional analysis, fatigue showed a strong association with depressive symptoms. Specifically, FACIT-F scores correlated significantly with WHO-5 (V1: r = 0.740, *p <* 0.001; V2: r = 0.801, *p <* 0.001) and PHQ-9 (V1: r = −0.795, *p <* 0.001; V2: r = −0.802, *p <* 0.001). Overall, the relationships between disease activity, function, and PROs observed at baseline persisted at one-year follow-up. Detailed results are shown in Tables 4, 5 and Figure 1.

**Table 4:** Disease activity.

Baseline total cohort Visit 1 (*n =* 238)	WHO-5	PHQ-9	EuroQoL-5D	FACIT
ASDAS	−0.310**	0.380**	−0.545**	−0.422**
BASDAI	−0.522**	0.607**	−0.601**	−0.643**
BASFI	−0.425**	0.507**	−0.696**	−0.551**
BASMI	−0.176*	0.136	−0.268**	−0.206**
FFbH	0.364**	−0.416**	0.645**	0.415**
Symptom duration (months)	−0.02	−0.083	−0.142*	−0.047

Follow-up. Visit 1 (*n =* 76)	WHO-5	PHQ-9	EuroQoL-5D	FACIT
ASDAS	−0.312**	0.362**	−0.646**	−0.454**
BASDAI	−0.443**	0.495**	−0.632**	−0.593**
BASFI	−0.300*	0.433**	−0.666**	−0.516**
BASMI	−0.101	0.124	−0.078	−0.183
FFbH	−0.388**	0.415**	−0.390**	−0.454**

Follow-up. Visit 2 (*n =* 76)	WHO-5	PHQ-9	EuroQoL-5D	FACIT
ASDAS	−0.372**	0.364**	−0.420**	−0.312**
BASDAI	−0.628**	0.625**	−0.606**	−0.612**
BASFI	−0.468**	0.652**	−0.648**	−0.534**
BASMI	−0.137	0.265*	−0.354**	−0.180
FFbH	0.438**	−0.503**	0.617**	0.394**
Symptom duration (months)	0.009	0.204	−0.203	−0.49

Functional status. Mobility and symptom duration at baseline and follow-up. Statistical significance levels in the tables are indicated as follows: *p < 0.05, **p < 0.01.

**Table 5:** Depressive symptoms, health-related quality of life and fatigue.

Baseline total cohort Visit 1 (*n =* 238)	WHO-5	PHQ-9	EuroQoL-5D	FACIT-F
WHO-5	1	−0.720**	0.382**	0.740**
PHQ-9	−0.720**	1	−0.448**	−0.795**
EuroQoL-5D	0.382**	−0.448**	1	0.503**
FACIT	0.740**	−0.795**	0,503**	1

Follow-up. Visit 1 (*n =* 76)	WHO-5	PHQ-9	EuroQoL-5D	FACIT-F
WHO-5	1	−0.737**	0.310**	0.719**
PHQ-9	−0.737**	1	−0.390**	−0.697**
EuroQoL-5D	0.310**	−0.390**	1	0.518**
FACIT	0.719**	−0.697**	0.518**	1

Follow-up. Visit 2 (*n =* 76)	WHO-5	PHQ-9	EuroQoL-5D	FACIT-F
WHO-5	1	−0.688**	0.481**	0.801**
PHQ-9	−0.688**	1	−0.576**	−0.804**
EuroQoL-5D	0.481**	−0,576**	1	0,539**
FACIT	0,801**	−0,804**	0,539**	1

Functional status. Mobility and symptom duration at baseline and follow-up. Statistical significance levels in the tables are indicated as follows: *p < 0.05, **p < 0.01.

### Predictors of depressive symptoms (PHQ-9, WHO-5)

Multiple linear regression analyses were performed to assess the influence of disease activity (BASDAI, ASDAS) and functional impairment (BASFI, FFbH) on depressive symptoms (PHQ-9, WHO-5)/ HRQoL (EuroQoL-5D)/ Fatigue (FACIT-F) at baseline and in the longitudinal one-year follow-up cohort (see Tables 6, 7).

**Table 6:** Multiple linear regression for PHQ-9 at baseline and one-year follow-up (Visit 1: total cohort *n =* 238; Visit 1: follow-up cohort *n =* 76; Visit 2: follow-up cohort *n =* 76).

Visit	Predictor	B	Beta	SE	*t*	*p*	95% CI
V1 - total cohort (*n =* 238)	Constant	−1.213	—	3.532	−0.344	0.732	−8.180–5.754
V1 - total cohort (*n =* 238)	FFbH	0.046	0.148	0.034	1.348	0.179	−0.021–0.114
V1 - total cohort (*n =* 238)	ASDAS	−1.723	−0.288	0.595	−2.896	*0.004*	−2.896–−0.549
V1 - total cohort (*n =* 238)	BASDAI	1.835	0.711	0.253	7.255	*<0.001*	1.336–2.334
V1 - total cohort (*n =* 238)	BASFI	0.702	0.316	0.280	2.503	*0.013*	0.149–1.255
V1 - follow-up (*n =* 76)	Constant	3.453	—	7.367	0.469	0.641	−11.295–18.201
V1 - follow-up (*n =* 76)	FFbH	0.037	0.109	0.073	0.516	0.608	−0.109–0.183
V1 - follow-up (*n =* 76)	ASDAS	−4.361	−0.719	1.334	−3.270	**0.002**	−7.032–−1.690
V1 - follow-up (*n =* 76)	BASDAI	2.412	0.908	0.657	3.673	*<0.001*	1.097–3.727
V1 - follow-up (*n =* 76)	BASFI	0.912	0.407	0.559	1.632	0.108	−0.207–2.031
V2 - follow-up (*n =* 76)	Constant	7.221	—	5.876	1.229	0.224	−4.559–19.002
V2 - follow-up (*n =* 76)	FFbH	−0.043	−0.153	0.057	−0.763	0.449	−0.157–0.070
V2 - follow-up (*n =* 76)	ASDAS	−1.216	−0.225	0.749	−1.623	0.110	−2.718–0.286
V2 - (follow-up *n =* 76)	BASDAI	1.185	0.508	0.430	2.759	*0.008*	0.324–2.046
V2 - follow-up (*n =* 76)	BASFI	0.543	0.258	0.477	1.139	0.260	−0.413–1.500

**Table 7:** Multiple linear regression for WHO-5 at baseline and one-year follow-up (Visit 1: total cohort *n =* 238; Visit 1: follow-up cohort *n =* 76; Visit 2: follow-up cohort *n =* 76).

Visit	Predictor	B	Beta	SE	*t*	*p*	95% CI
V1 - total cohort (*n =* 238)	Constant	62.102	—	17.590	3.531	<0.001	27.397–96.806
V1 - total cohort (*n =* 238)	FFbH	0.018	0.012	0.172	0.103	0.918	−0.321–0.356
V1 - total cohort (*n =* 238)	ASDAS	9.617	0.344	2.899	3.318	*0.001*	3.898–15.336
V1 - total cohort (*n =* 238)	BASDAI	−8.803	−0.723	1.260	−6.989	*<0.001*	−11.288–−6.318
V1 - total cohort (*n =* 238)	BASFI	−1.599	−0.153	1.410	−1.134	0.258	−4.380–1.183
V1 - follow-up (*n =* 76)	Constant	31.440	—	37.014	0.849	0.399	−42.768–105.648
V1 - follow-up (*n =* 76)	FFbH	0.214	0.144	0.366	0.584	0.561	−0.520–0.948
V1 - follow-up (*n =* 76)	ASDAS	17.825	0.676	6.429	2.773	**0.008**	4.936–30.714
V1 - follow-up (*n =* 76)	BASDAI	−12.722	−1.094	3.190	−3.989	*<0.001*	−19.118–−6.326
V1 - follow-up (*n =* 76)	BASFI	1.945	0.202	2.795	0.696	0.489	−3.659–7.549
V2 - follow-up (*n =* 76)	Constant	43.790	—	34.534	1.268	0.210	−25.418–112.999
V2 - follow-up (*n =* 76)	FFbH	0.358	0.221	0.334	1.070	0.289	−0.312–1.028
V2 - follow-up (*n =* 76)	ASDAS	6.463	0.210	4.471	1.446	0.154	−2.497–15.423
V2 - (follow-up *n =* 76)	BASDAI	−12.225	−0.916	2.568	−4.760	*<0.001*	−17.372–−7.078
V2 - follow-up (*n =* 76)	BASFI	4.053	0.337	2.819	1.438	0.156	−1.596–9.702

At baseline in the total cohort, BASDAI (*β* = 0.711, *p <* 0.001), ASDAS (*β* = −0.288, *p =* 0.004), and BASFI (*β* = 0.316, *p =* 0.013) were significant predictors of PHQ-9, whereas FFbH was not statistically significant. In the subgroup of 76 patients who were available for follow-up after 1 year, only BASDAI and ASDAS appeared as strong predictors at baseline. At the one-year follow-up, BASDAI remained the only significant predictor (*β* = 0.508, *p =* 0.008), while the remaining variables did not reach statistical significance (Tables 8, 9).

**Table 8:** Multiple linear regression for EuroQoL-5D at baseline and one-year follow-up (Visit 1: total cohort *n =* 238; Visit 1: follow-up cohort *n =* 76; Visit 2: follow-up cohort *n =* 76).

Visit	Predictor	B	Beta	SE	*t*	*p*	95% CI
V1 - total cohort (*n =* 238)	Constant	0.616	—	0.181	3.406	<0.001	0.259–0.973
V1 - total cohort (*n =* 238)	FFbH	0.004	0.245	0.002	2.293	**0.023**	0.001–0.008
V1 - total cohort (*n =* 238)	ASDAS	−0.035	−0.112	0.030	−1.138	0.257	−0.094–0.025
V1 - total cohort (*n =* 238)	BASDAI	−0.009	−0.068	0.013	−0.699	0.485	−0.035–0.017
V1 - total cohort (*n =* 238)	BASFI	−0.036	−0.310	0.014	−2.525	**0.012**	−0.065–−0.008
V1 - follow-up (*n =* 76)	Constant	0.775	—	0.371	2.090	0.041	0.032–1.518
V1 - follow-up (*n =* 76)	FFbH	0.003	0.169	0.004	0.777	0.440	−0.005–0.011
V1 - follow-up (*n =* 76)	ASDAS	−0.077	−0.260	0.066	−1.167	0.248	−0.209–0.055
V1 - follow-up (*n =* 76)	BASDAI	0.005	0.037	0.033	0.147	0.883	−0.061–0.071
V1 - follow-up (*n =* 76)	BASFI	−0.032	−0.295	0.028	−1.162	0.250	−0.088–0.024
V2 - follow-up (*n =* 76)	Constant	−0.249	—	0.227	−1.096	0.278	−0.706–0.207
V2 - follow-up (*n =* 76)	FFbH	0.012	0.948	0.002	5.623	**<0.001**	0.008–0.017
V2 - follow-up (*n =* 76)	ASDAS	0.006	0.025	0.032	0.194	0.847	−0.059–0.071
V2 - (follow-up *n =* 76)	BASDAI	−0.013	−0.122	0.018	−0.718	0.476	−0.050–0.023
V2 - follow-up (*n =* 76)	BASFI	0.024	0.244	0.019	1.255	0.215	−0.014–0.062

**Table 9:** Multiple linear regression for FACIT-F at baseline and one-year follow-up (Visit 1: total cohort *n =* 238; Visit 1: follow-up cohort *n =* 76; Visit 2: follow-up cohort *n =* 76).

Visit	Predictor	B	Beta	SE	*t*	*p*	95% CI
V1 - total cohort (*n =* 238)	Constant	59.905	—	6.975	8.589	<0.001	46.146–73.664
V1 - total cohort (*n =* 238)	FFbH	−0.140	−0.213	0.068	−2.066	*0.040*	−0.275–−0.006
V1 - total cohort (*n =* 238)	ASDAS	2.871	0.230	1.165	2.465	**0.015**	0.574–5.169
V1 - total cohort (*n =* 238)	BASDAI	−3.756	−0.688	0.504	−7.456	*<0.001*	−4.749–−2.762
V1 - total cohort (*n =* 238)	BASFI	−1.880	−0.400	0.559	−3.365	*<0.001*	−2.982–−0.778
V1 - follow-up (*n =* 76)	Constant	44.096	—	14.723	2.995	0.004	14.625–73.567
V1 - follow-up (*n =* 76)	FFbH	−0.006	−0.008	0.145	−0.039	0.969	−0.296–0.284
V1 - follow-up (*n =* 76)	ASDAS	3.162	0.262	2.665	1.186	0.240	−2.173–8.497
V1 - follow-up (*n =* 76)	BASDAI	−3.865	−0.732	1.312	−2.946	**0.005**	−6.492–−1.238
V1 - follow-up (*n =* 76)	BASFI	−0.676	−0.152	1.117	−0.605	0.547	−2.912–1.560
V2 - follow-up (*n =* 76)	Constant	34.517	—	13.645	2.530	0.014	7.171–61.863
V2 - follow-up (*n =* 76)	FFbH	0.105	0.171	0.132	0.791	0.432	−0.160–0.369
V2 - follow-up (*n =* 76)	ASDAS	2.769	0.238	1.767	1.567	0.123	−0.771–6.309
V2 - (follow-up *n =* 76)	BASDAI	−3.673	−0.731	1.015	−3.619	*<0.001*	−5.707–−1.639
V2 - follow-up (*n =* 76)	BASFI	0.347	0.077	1.114	0.311	0.757	−1.885–2.579

For WHO-5, baseline analyses in both the total cohort and the subgroup with available one-year follow-up data identified BASDAI (*β* = −0.723, *p <* 0.001) and ASDAS (*β* = 0.344, *p =* 0.001) as significant predictors, whereas BASFI and FFbH did not show significant associations. At the one-year follow-up, BASDAI remained the sole significant predictor (*β* = −0.916, *p <* 0.001).

These findings indicate that BASDAI consistently predicts depressive symptoms and wellbeing, highlighting that higher patient-reported disease activity is closely associated with worse mental health outcomes in axSpA, whereas physician-assessed disease activity (ASDAS) and functional measures show smaller or non-significant effects over time.

### Predictors of health-related quality of life (EuroQoL-5D)

At baseline, FFbH (*β* = 0.245, *p =* 0.023) and BASFI (*β* = −0.310, *p =* 0.012) were significant predictors of EQ-5D, whereas BASDAI and ASDAS were not. In contrast, baseline analyses within the subgroup with available follow-up data yielded a significant overall regression model, although none of the individual predictors demonstrated an independent significant association with EQ-5D, suggesting substantial shared variance among the included clinical measures. At follow-up, FFbH remained the only significant predictor of HRQoL (*β* = 0.948, *p <* 0.001), with other variables not reaching statistical significance.

Overall, these findings suggest that functional capacity (FFbH) is the most consistent predictor of HRQoL, with higher functional performance associated with better HRQoL, whereas disease activity (BASDAI, ASDAS) showed no independent association in this cohort.

### Predictors of fatigue (FACIT-F)

At baseline in the overall cohort, FFbH (*β* = −0.213, *p =* 0.040), ASDAS (*β* = 0.230, *p =* 0.015), BASDAI (*β* = −0.688, *p <* 0.001), and BASFI (*β* = −0.400, *p <* 0.001) were significant predictors of FACIT-F. In contrast, baseline analyses in the subgroup with available follow-up data identified BASDAI as the only significant predictor (*β* = −0.732, *p =* 0.005). At the one-year follow-up, BASDAI remained the sole significant predictor (*β* = −0.731, *p <* 0.001), whereas all other variables failed to reach statistical significance.

## Summary

Overall, BASDAI consistently predicted depressive symptoms, reduced mental well-being, and fatigue, while FFbH was the strongest predictor of HRQoL. Functional impairment (BASFI) and physician-assessed disease activity (ASDAS) showed smaller or time-limited effects.

## Discussion

Rheuma-VOR is one of the few longitudinal studies assessing mental health, HRQoL, and fatigue in patients with axSpA both prior to the initiation of adequate treatment and after 1 year of follow-up (21, 22). Another distinguishing feature of Rheuma-VOR is the earlier diagnosis, with a mean of only 3.92 years, which is substantially lower than the German average of 8.4 years as reported by the German Rheumatism Research Center (DRFZ) (9). Following diagnosis, patients were initiated on guideline-conform first-line therapy for axSpA in accordance with contemporary ASAS-EULAR recommendations. Treatment response, disease control, and remission status were prospectively monitored over the one-year follow-up by board-certified rheumatologists, with therapy escalation applied according to guideline-based treat-to-target principles whenever clinically indicated (2, 9, 22, 23).

Patients’ improvements in mental health, HRQoL and fatigue were closely associated with reductions in overall disease burden, while worsening outcomes were observed in parallel with higher disease burden. Symptom duration did not correlate with disease activity or any other outcome, likely due to the unusually short time to diagnosis. Compared with disease activity, sociodemographic variables showed a weaker association with these outcomes. Patient-reported disease burden was the strongest predictor of psychological distress and fatigue, whereas at baseline impairments in HRQoL were primarily associated with functional limitations.

### Mental health

The Rheuma-VOR findings are consistent with international evidence indicating a higher prevalence of depressive symptoms in patients with axSpA compared with the general population (34–36). A meta-analysis by Zhao et al. (37) reported a pooled prevalence of mild depressive symptoms of approximately 38%. Direct comparisons across studies remain limited due to heterogeneity in assessment instruments and timing of evaluation. Similar prevalence estimates were reported in an Indian axSpA cohort, in which 36% of patients exhibited depressive symptoms based on the PHQ-9. In that cohort, depressive symptoms were strongly associated with higher disease activity (BASDAI > 4), fatigue (FACIT-F), and psychological distress. In contrast to Rheuma-VOR, female sex and younger age at disease onset were additional risk factors. Moreover, depressive symptoms were assessed approximately 5 years after diagnosis, limiting comparability with the early disease stage examined in Rheuma-VOR (15). Other international cohort studies confirm a robust association between disease burden and depressive symptoms, although prevalence rates vary considerably. In a Turkish cohort, female sex was identified as a risk factor for depressive symptoms (38). Conversely, a Greek study conducted at a later disease stage and including a predominantly male population (85.5%) reported lower prevalence rates, with 14.8% of patients meeting criteria for major depression and 25.9% presenting with mild depressive symptoms (39). Differences in sex distribution, disease duration, and sociocultural factors may contribute to these discrepancies (40, 41).

German data reported by Redeker et al. (42) demonstrated prevalence rates of reduced wellbeing assessed by the WHO-5 comparable to those observed in Rheuma-VOR, with approximately half of patients reporting at least mild depressive symptoms. Unlike Rheuma-VOR, depressive symptoms in that cohort were associated with socioeconomic status, body mass index, and smoking status, and were accompanied by a higher burden of comorbidities.

Overall, the Rheuma-VOR data indicate that psychological distress is common and clinically relevant in axSpA, particularly at the time of diagnosis. The observed improvement in depressive symptoms during the first year of follow-up suggests that early and adequate treatment may positively influence mental health outcomes alongside disease activity. Although the PHQ-9 and WHO-5 do not replace formal psychiatric assessment, both are internationally validated tools that allow comparability across cohorts. Rheuma-VOR therefore provides important longitudinal data, as few studies have assessed mental health at diagnosis and during early follow-up.

### Fatigue

Fatigue is a prevalent and burdensome symptom in axSpA and is widely considered a marker of disease activity, as reflected by its inclusion in the BASDAI (43). In the Rheuma-VOR study, fatigue was assessed as a distinct and differentiated outcome to enable a more comprehensive understanding of fatigue beyond the context of disease activity. This approach was chosen because fatigue may be influenced by a range of additional factors, including patient age, comorbidities, and occupational demands, and should therefore be considered, at least in part, independently of disease status. 70% of patients in the Rheuma-VOR cohort reported at least mild fatigue at diagnosis. After 1 year of specialized rheumatological care, the prevalence decreased to 58.7%; however, approximately half of patients continued to report persistent mild fatigue. Disease activity, as measured by the BASDAI, was the strongest predictor of fatigue.

As fatigue is a component of the BASDAI, these associations should be interpreted cautiously. Nevertheless, multiple international studies have demonstrated a close relationship between fatigue and disease activity. In the French DESIR cohort, which also assessed patients at an early disease stage, fatigue levels were similarly high (44). DESIR, COSPA, and several other cohorts, fatigue was primarily assessed using numerical rating scales rather than validated multidimensional instruments (44–46). One of the few studies applying the FACIT-F alongside BASDAI and BASFI reported a strong correlation between BASDAI and FACIT-F scores, although the sample size was limited (32).

Rheuma-VOR is among the few longitudinal studies to evaluate treatment effects in axSpA using multiple validated patient-reported outcome measures assessed before and after therapy initiation. However, the one-year follow-up cohort was relatively small (*n =* 76), and the observation period was limited. As comorbidities and functional impairments may evolve over longer periods, these outcomes may not have been fully captured.

## Conclusion

In conclusion, disease activity, fatigue, and mental health are closely interrelated in axSpA patients. Higher disease activity, particularly as measured by the BASDAI, is associated with increased fatigue and depressive symptom burden, whereas improvements in physical disease parameters coincide with improvements in psychological wellbeing. These findings support early and systematic screening for depressive symptoms in axSpA and highlight the need for interdisciplinary management approaches addressing both physical and mental health aspects of the disease.

## Data Availability

The original contributions presented in the study are included in the article/supplementary material.
